# Rapid Characterization of Microalgae and Microalgae Mixtures Using Matrix-Assisted Laser Desorption Ionization Time-Of-Flight Mass Spectrometry (MALDI-TOF MS)

**DOI:** 10.1371/journal.pone.0135337

**Published:** 2015-08-13

**Authors:** Duane Barbano, Regina Diaz, Lin Zhang, Todd Sandrin, Henri Gerken, Thomas Dempster

**Affiliations:** 1 School of Life Sciences, Arizona State University, 427 East Tyler Mall, Tempe, Arizona, United States of America; 2 School of Mathematical and Natural Sciences, Arizona State University, MC 2352, P.O. Box 37100, Phoenix, Arizona, United States of America; 3 Arizona Center for Algae Technology and Innovation, Arizona State University, 7418 Innovation Way South, Building ISTB-3, Room 103, Mesa, Arizona, United States of America; The George Washington University, UNITED STATES

## Abstract

Current molecular methods to characterize microalgae are time-intensive and expensive. Matrix Assisted Laser Desorption/Ionization Time-of-Flight Mass Spectrometry (MALDI-TOF MS) may represent a rapid and economical alternative approach. The objectives of this study were to determine whether MALDI-TOF MS can be used to: 1) differentiate microalgae at the species and strain levels and 2) characterize simple microalgal mixtures. A common protein extraction sample preparation method was used to facilitate rapid mass spectrometry-based analysis of 31 microalgae. Each yielded spectra containing between 6 and 56 peaks in the m/z 2,000 to 20,000 range. The taxonomic resolution of this approach appeared higher than that of 18S rDNA sequence analysis. For example, two strains of *Scenedesmus acutus* differed only by two 18S rDNA nucleotides, but yielded distinct MALDI-TOF mass spectra. Mixtures of two and three microalgae yielded relatively complex spectra that contained peaks associated with members of each mixture. Interestingly, though, mixture-specific peaks were observed at m/z 11,048 and 11,230. Our results suggest that MALDI-TOF MS affords rapid characterization of individual microalgae and simple microalgal mixtures.

## Introduction

Microalgae have received considerable attention in science and industry as they can be cultivated and harvested for many products and co-products including biofuels and nutraceuticals [1]. Microalgae have different growth rates which are affected by a range of environmental factors such as nutrient availability and temperature. Those environmental factors need to be controlled in order to generate product, especially in large-scale biomass production [2]; however, the environmentally-exposed open pond system model leaves microalgae cultures susceptible to contamination by undesired microalgae that can out-compete the original microalga for resources, which can negatively affect production [2][3]. This shift in microalgae species can go unnoticed if the species are phenotypically similar. As a result, microalgae in mass-production systems need to be monitored regularly for contamination to avoid a decrease in productivity and catastrophic culture crashes.

Conventional techniques for microalgae identification include morphological analysis using bright field light microscopy and electron microscopy [4]. Complementary molecular techniques include multilocus sequence typing (MLST) [5], repetitive sequence-based polymerase chain reaction (rep-PCR) [6], 18S rDNA analysis [7], and pulsed-field gel electrophoresis (PFGE) [8]. In many instances, the use of these techniques requires amounts of time, labor, and resources that are impractical [9] for monitoring the health of microalgae ponds in near real-time.

Matrix-assisted laser desorption/ionization time-of-flight mass spectrometry (MALDI-TOF MS) is a technique that has been shown capable of rapidly and reliably characterizing bacteria at the genus, species, and in some cases, strain levels [10] and is becoming more routine in use [11]. Most often, this is achieved by comparing mass spectra (i.e., fingerprints) acquired from crude protein extracts of unknown microorganisms to reference spectra in databases [12]. Furthermore, studies have shown that MALDI-TOF MS-based fingerprint methods may afford greater taxonomic resolution than traditional molecular techniques [9][10][13]. In addition to bacteria, MALDI-TOF MS has also been used to characterize fungi [14–17], viruses [18], and more recently to a considerably lesser extent, microalgae [4][5][19][20]. Nicolau and colleagues [4] obtained spectra of diatoms using MALDI-TOF MS and observed that culture age affected mass spectra. Von Bergen et al. [21] used MALDI-TOF MS to characterize five pathogenic species of *Prototheca*, and Wirth et al. [22][23] showed that optimization of downstream analyses such as self-organizing mapping (SOM portrait analysis) of spectra allowed MALDI-TOF MS to discriminate between harmless and pathogenic *Prototheca* species. Most recently, Emami et al. [20] obtained greater taxonomic resolution during characterization of 31 strains of *Dunaliella* sp. with MALDI TOF MS than with internal transcribed spacer (ITS) sequence analysis. Each of these studies suggests that MALDI-TOF MS has promise as a tool for the rapid characterization of diverse, economically-relevant microalgae [24][25].

To further explore the ability of MALDI-TOF MS to characterize microalgae, we focused on 31 algae representing 12 species. The specific objectives of this study were to determine whether MALDI-TOF MS can be used: 1) for species-level differentiation of economically-relevant algae; 2) for strain-level characterization; and 3) to characterize simple mixtures of microalgae. A common protein extraction sample preparation method was used. Sequence (18S rDNA) analysis was performed on all microalgae to confirm their identity and to compare the taxonomic resolution afforded by this traditional approach to a MALDI-based approach. Finally, two model mixture systems containing two and three microalgae were examined. Our results suggest that MALDI-TOF MS affords rapid: 1) characterization of a diverse collection of microalgae, 2) discrimination between multiple strains within a single species, and 3) characterization of simple mixtures.

## Materials and Methods

### Reagents

MALDI matrix, α-cyano-4-hydroxycinnaminic acid (CHCA), and trifluoroacetic (TFA) acid were purchased from ACROS (Fair Lawn, NJ, USA). Acetonitrile (ACN) was purchased from Alfa Aesar (Ward Hill, MA, USA). MALDI calibrants (ACTH 1–17 (2,093.46 Da), ACTH 18–39 (2,464.19 Da), Insulin Oxidized B 3,494.65 Da), Insulin (5,730.61 Da), Cytochrome C (12,362.00 Da), and Apomyoglobin (16,952.30 Da)) and formic acid (FA) were purchased from Sigma (St. Louis, MO, USA). Ultrapure water was generated using a Milli-Q integral water purification system (Millipore Corporation, Billerica, MA, USA).

### Microalgae Cultivation

Thirty-one microalgae representing 10 genera and 12 species were provided by the Arizona Center for Algae Technology and Innovation (AzCATI; http://www.AzCATI.com) (Table 1). Specifically, six of the genera were freshwater species (*Chlamydomonas*, *Chlorella*, *Parachlorella*, *Chromochloris*, *Desmodesmus* and *Scenedesmus*), and five were marine species (*Dunaliella*, *Chlorella*, *Tetraselmis*, *Nannochloropsis*, and *Porphyridium*). Five mL of BG-11 [26] for freshwater strains or F/2 [27] for marine strains were inoculated with a single colony of microalgae growing axenically on petri plates aseptically in a laminar flow hood. Microalgae samples were grown in 15 mL screwcap tubes with 75 μmol/m^2^/s of cool white fluorescent lighting at 20°C for 3 weeks prior to analysis with MALDI-TOF MS.

**Table 1:** Microalgae species and strains used in this study.

Sample	Genus	Species	Strain	Medium	Genbank Accession Number
1	*Chlorella*	*vulgaris*	UTEX 395	Freshwater	KR904898
2	*Chlorella*	*vulgaris*	UTEX 259	Freshwater	KR904897
3	*Chlorella*	*vulgaris*	LRB-AZ 1201	Freshwater	KR904896
4	*Chlorella*	*sorokiniana*	UTEX 2714	Freshwater	LK021940.1
5	*Parachlorella*	*kessleri*	CBS 15–2069^1^	Freshwater	KR904906
6	*Chromochloris*	*zofingeinsis*	UTEX 32	Freshwater	KR904902
7	*Chromochloris*	*zofingeinsis*	LRB-AZ 701	Freshwater	KR904901
8	*Chlorella*	*sorokiniana*	UTEX 1230	Freshwater	KR904895
9	*Chlamydomonas*	*reinhardtii*	CC 849	Freshwater	KR904894
10	*Chlamydomonas*	*reinhardtii*	CBS 15–2030	Freshwater	KR904892
11	*Desmodesmus*	*abundans*	LRB-CO 801	Freshwater	KR904903
12	*Chlamydomonas*	*reinhardtii*	CBS 15–2280^2^	Freshwater	KR904893
13	*Scenedesmus*	*acutus*	LRB-AP 401	Marine	KR904911
14	*Scenedesmus*	*acutus*	LRB-AZ 414	Marine	KR904912
15	*Dunaliella*	*salina*	CBS 15–2160	Marine	KR904904
16	*Nannochloropsis*	*gaditana*	CCMP 526	Marine	KF040086.1
17	*Nannochloropsis*	*gaditana*	CCMP 527	Marine	AF045038.1
18	*Nannochloropsis*	*salina*	CCMP 1776	Marine	KJ756828.1
19	*Nannochloropsis*	*salina*	CCMP 537	Marine	AF045049.1
20	*Nannochloropsis*	*granulata*	CCMP 529	Marine	U41092.1
21i	*Chlorella*	sp.	LRB-AZ 1221	Marine	KR904899
22	*Nannochloropsis*	*limnetica*	CCMP 505	Marine	U41050.1
23	*Nannochloropsis*	*granulata*	CCMP 525	Marine	AF045044.1
24	*Nannochloropsis*	*oceanica*	CCMP 531	Marine	U41094.1
25	*Nannochloropsis*	*oceanica*	IMET-1	Marine	KR904905
26	*Nannochloropsis*	*oceanica*	CCAP 849/10	Marine	KJ756836.1
27	*Porphyridium*	*purpureum*	CBS 15–3599	Marine	KR904907
28	*Porphyridium*	*purpureum*	LRB-OH 6101	Marine	KR904908
29	*Tetraselmis*	sp.	CBS 15–2475	Marine	KR904909
30	*Tetraselmis*	sp.	CBS 15–2610	Marine	KR904910
31	*Chlorella*	*vulgaris*	LRB-FL 1220	Marine	KR904900

^1^ Carolina 152069 was identified by Carolina Biological as *Chlorella spp*.

^2^ Carolina 152280 was identified by Carolina Biological as *Haematococcus spp*.

### Sample Preparation for MALDI-TOF MS

A common, previously described protein extraction procedure was used as the basis for the sample preparation method used here [28]. One mL of cells at an optical density of 750 nm (OD_750_) between 0.15 and 0.3 were washed with sterile milliQ-H_2_O (mQ-H_2_O) and then inactivated for 1 hour in 300 μL mQ-H_2_O and 900 μL absolute ethanol. Samples were then centrifuged at 10,000 x g for two minutes at room temperature. The supernatant was decanted, and the cells were resuspended in 1 ml mQ-H_2_O, centrifuged at 10,000 x g for two minutes once more, and the supernatant was again decanted. FA and ACN were added to the resulting pellet. Equal volumes of FA and ACN were added in volumes necessary to normalize to an initial culture OD_750_ = 0.8. Pellets were vortexed vigorously. The samples were then centrifuged at 17,000 xg for five minutes at room temperature, and the supernatant was collected and used immediately for MALDI analysis. Triplicate 1-μL aliquots of each supernatant were plated onto a MSP 96 Polished Steel MALDI Target Plate (Bruker Daltonics, Billerica, MA, USA) and allowed to air dry. A CHCA matrix solution was prepared by mixing 25 μL 99.5% TFA, 500 μL ACN, 475 μL mQ-H_2_O, and 15 mg CHCA. Each sample was covered with 1 μL CHCA matrix solution and allowed to air dry.

### Mass Spectra Acquisition

A Bruker Microflex LRF MALDI-TOF MS (Bruker Daltonics) was used to acquire mass spectra. The spectrometer was equipped with a 337 nm nitrogen laser and controlled using FlexControl software (version 3.0; Bruker Daltonics). Mass spectra in the m/z 2,000 to 20,000 range were collected automatically in the positive linear mode. Ion source 1 was set to 20 kV, and ion source 2 was set to 18.15 kV with the lens set to 9.05 kV. Spectra for each sample were generated from 500 laser shots acquired in five 100 shot bursts. The laser frequency was set to 10 Hz. Spectra from each of the 100 shot bursts were included only if the following parameters were met: a base peak (i.e., the peak with the greatest intensity) signal-to-noise ratio (S:N) of 2 or greater, a peak width of 10 m/z, a minimum intensity threshold of 100, and a maximal number of peaks of 500. Three replicate MALDI mass spectra were obtained per algal strain. Peak smoothing was performed using the Savitzky-Golay algorithm. Baseline subtraction was performed using the TopHat algorithm. Calibration of the mass spectrometer was performed using a protein calibrant mixture containing the proteins listed above. Peaks were identified using FlexAnalysis 3.0 software (Bruker Daltonics) and then transferred to a Microsoft Excel spreadsheet in which the average mass range and base peak signal-to-noise ratios were calculated to assess spectrum quality. Peaks were considered different if they varied by more than +/- 2 m/z.

### MALDI-TOF MS Data Analysis

Additional analysis of spectra was performed using BioNumerics (v. 7; Applied Maths, Austin, TX, USA). Composite spectra (i.e., summary spectra) were created using data from all replicates to represent each of the 31 microalgae. A similarity threshold of 65% was used to ensure representation of each replicate spectrum in the summary spectra. Pseudo-gels were constructed to visualize the MALDI profiles for each microalga. Similarity was quantified using the Pearson correlation coefficient, and a dendrogram was generated by using the UPGMA method.

### 18S rDNA Sequence Analysis

The genomic 18S ribosomal DNA region of microalgae was amplified by colony PCR as described previously [29] using the 360FE: 5’-CGGAGARGGMGCMTGAGA-3’ [30] forward and 26R-1: 5’-GTTAGTTTCTTTTCCTCCGC-3’ [30] reverse primers. Following amplification, PCR products were electrophoresed on a 0.8% agarose gel and purified using a Zymoclean DNA gel recovery kit (Zymo Research, Irvine, CA). Sequencing was performed at the ASU Core DNA sequencing facility on 20 ng of template using the primers 360FE and 1391RE: 5’-GGGCGGTGTGTACAARGRG-3’ described previously [30] and the following primers constructed for this study: 18SF3 5’-GTCAGAGGTGAAATTCTTGG, 18SF4 5’-CGGCTTAATTTGACTCAACACGGG-3’, 18SR2 5’-AAGAACGGCCATGCACCACCACCC-3’, 18SR3 5’-CCCAACTTTCGTTCTTGATTAATG-3’.

Analysis of the 18S rDNA sequences for the 31 microalgae samples was performed using BioNumerics (v. 7; Applied Maths, Austin, TX, USA). The similarity coefficient was multiple-alignment based with a Kimura correction and 12% gap penalty. The dendrogram build method was neighbor joining. No out group was used in the construction of the dendrogram.

### Characterization of mixtures of microalgae using MALDI-TOF MS

Two model mixture systems were constructed using samples composed of two or three microalgae cultures. The OD_750_ for each microalga was adjusted to 0.3 before mixing so that the microalgae would be represented equally in the mixture. The first mixed culture contained two microalgae, *Chlorella vulgaris* UTEX 395 and *Scenedesmus acutus* LRB-AP 401. To construct the mixture, 500 μL of each culture were added to a microcentrifuge tube to yield a 1 mL solution. The second mixed culture contained three microalgae: *C*. *vulgaris* UTEX 395, *S*. *acutus* LRB-AP 401, and *Chlorella sorokiniana* UTEX 1230. The 1 mL mixture was constructed using 333 μL of each microalga. Samples were prepared for MALDI analysis, and mass spectra were acquired using the procedures described above. Peak lists of the three individual samples and the two mixtures were compared. Each peak was included in the peak list only if it was present in all replicates. Peak matching was performed to identify prominent peaks attributed to individual isolates and peaks that were mixture-specific.

## Results and Discussion

### MALDI-TOF Spectra of Microalgae

**Fig 1:**
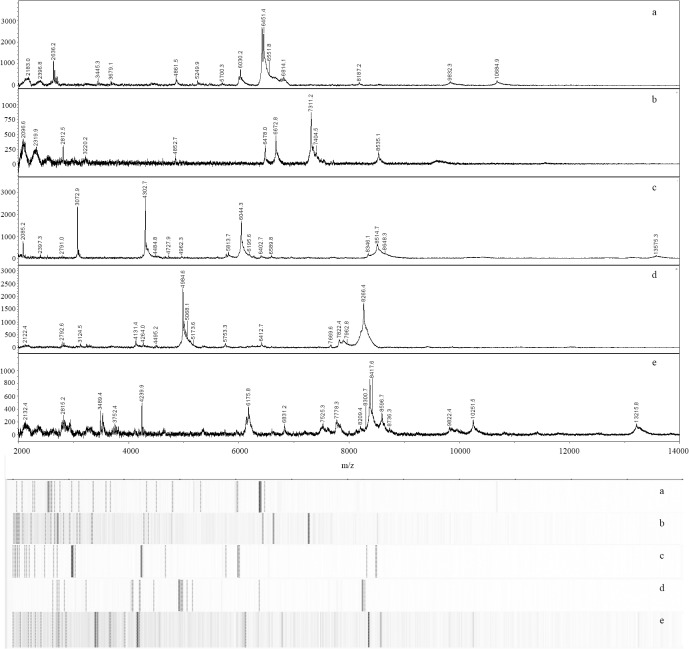
MALDI-TOF spectra and pseudo-gels of members of the five microalgae classes included in this study. Spectra and corresponding pseudo-gels of *Chlorella vulgaris* UTEX 395 (Trebouxphyceae) (a), *Porphyridium purpureum* LRB-OH 6101 (Porphyridiophyceae) (b), *Tetraselmis spp*. CBS 15–2610 (Chlorodendrophyceae) (c), *Chlamydomonas reinhardtii* CC 849 (Chlorophyceae) (d), and *Nannochloropsis oceanica* IMET-1 (Eustimatophyceae) (e).

MALDI-TOF MS yielded unique spectra for each microalga in the collection. We examined a mass range of m/z 2,000–20,000. A recent study [19] utilized a slightly narrower (m/z 4,000–20,000) range when characterizing microalgae via MALDI. The broader mass range we employed contained peaks that appeared useful for microalgae characterization. For example, several peaks below m/z 4,000 were observed in the spectra of multiple genera including *Chlorella vulgaris* UTEX 395 (Fig 1A), *Porphyridium purpureum* LRB-OH 6101 (Fig 1B), *Tetraselmis sp*. CBS 15–2610 (Fig 1C), *Chlamydomonas reinhardtii* CC 849 (Fig 1D), and *Nannochloropsis oceanica* IMET-1 (Fig 1E).

Mass ranges and peak numbers varied among the spectra of microalgae examined here. Masses in the spectrum of *C*. *vulgaris* UTEX 395 ranged from m/z 2,044 to 10,685. Comparatively, spectra of *P*. *purpureum* LRB-OH 6101, *Tetraselmis sp*. CBS 15–2610, and *C*. *reinhardtii* CC 849 exhibited narrower mass ranges (as low as m/z 2,005 and up to m/z 8,701 with *Tetraselmis* sp.). Spectra of *N*. *oceanica* IMET-1 had the broadest mass range (m/z 2,087 to 13,265). Numbers of peaks for the samples described here ranged from 6 for *Chromochloris zofingeinsis* UTEX 32 up to 56 peaks for *Nannochloropsis granulata* CCMP 525. These results are comparable to the work of Lee et al. [7], who used MALDI-TOF MS to characterize *Nannochloropsis granulata*, *Chlorella* sp., and *Dunaliella* sp. While spectra shown here are not identical to those described previously (e.g., Lee et al. [7] reported a broader mass range with *Chlorella* as well as a prominent peak near m/z 8,700 not observed in our spectra), both our work and that of Lee et al. [7] suggests that MALDI affords rapid and clear differentiation of diverse microalgae.

**Fig 2:**
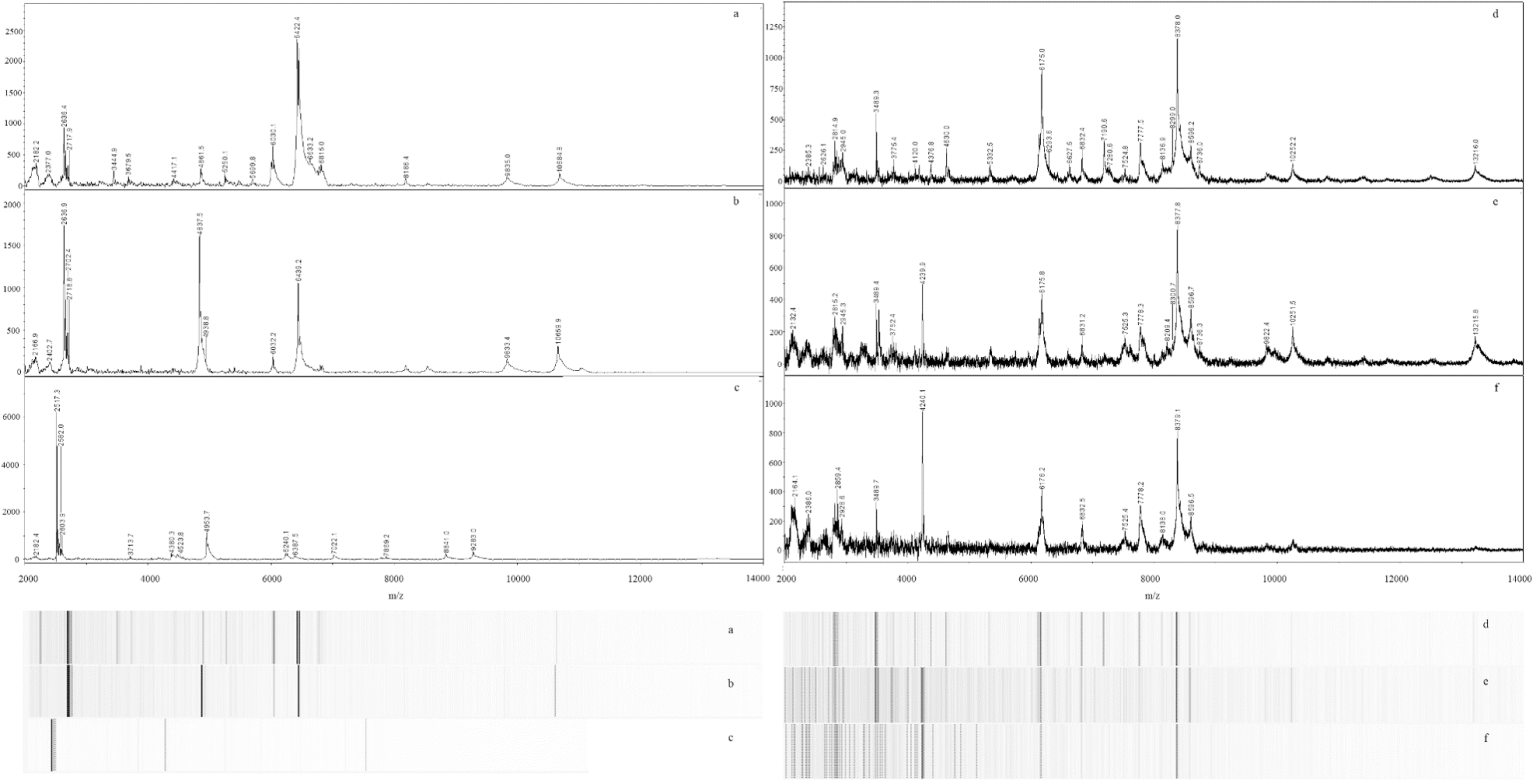
Representative MALDI-TOF spectra and pseudo-gels of microalgae strains of the same species. Three *C*. *vulgaris* spectra (a–c) exhibit strain-level differences: UTEX 395 (a), UTEX 259 (b), and LRB-AZ 1201 (c). Three *N*. *oceanica* spectra (d–f) also exhibit strain-level differences: CCMP 531 (d), IMET-1 (e), and CCAP 849/10 (f).

Moving beyond species-level characterization, we examined the capability of MALDI to characterize microalgae at the strain-level. The works of Murugaiyan [23], von Bergen [21], and Wirth [22] using members of the genus *Prototheca* support the ability of MALDI to distinguish between strains of microalgae within the same species. Our data suggest that strain-level differentiation of members of the genus *Chlorella* is feasible. Mass spectra of three *Chlorella* strains (*C*. *vulgaris* UTEX 395, *C*. *vulgaris* UTEX 259, and *C*. *vulgaris* LRB-AZ 1201) are clearly distinct (Fig 2A–2C). Spectra of all three microalgae exhibited similar mass ranges of m/z 2,182–10,685; 2,167–10,660; and 2,182–9,283, respectively; however, the spectra contained different base peaks at m/z 6,422; 2,637; and 2,517, respectively. These distinct spectra may be explained, in part, by the work of Gerken et al., who demonstrated previously that while the 18S rDNA sequences between 11 *C*. *vulgaris* strains were over 99% similar, the sensitivity of each strain to specific enzymes was remarkably distinct, indicating a highly variable cell wall composition among the various strains [29].

Similar to our results with *C*. *vulgaris*, spectra of three *Nannochloropsis* strains (*N*. *oceanica* CCMP 531, *N*. *oceanica* IMET-1, and *N*. *oceanica* CCAP 849/10) appeared to afford strain-level differentiation (Fig 2D–2F). Spectra of all three of these strains contained a characteristic peak near m/z 8,378. The spectrum of strain CCAP 849/10 contained a different base peak (m/z 4,240) than the other two *N*. *oceanica* strains. Additional differences in peaks among spectra of these strains were observed (Fig 2D–2F).

Most recently, Emami et al. [20] have reported results similar to ours in which MALDI-TOF MS appeared to afford greater taxonomic resolution in microalgae than gene sequence-based methods. In particular, they were able to differentiate strains of *Dunaliella*. Similar to our work, they also used a mass range of m/z 2,000 to 20,000. Our work is comparable in size (i.e., number of isolates analyzed), but broader (i.e., focus beyond a single genus) in taxonomy compared to the work of Emami et al. Interestingly, Emami et al. reported that whole cell-based sample preparation was necessary to yield useful spectra [20]. Our results, however, suggest that a relatively common, protein extraction-based approach to sample preparation is sufficient to produce MALDI spectra of microalgae that yield species- and strain-level characterization. While spectra we report here and those reported by Emami [20] for *C*. *vulgaris* are not identical, prominent peaks below m/z 3,000 are observed in spectra produced by both groups. Differences between spectra are likely related to different sample preparations, different strains, and differences in life stages used in each study.

### Comparison of 18S rDNA Sequence and MALDI-TOF Data

**Fig 3:**
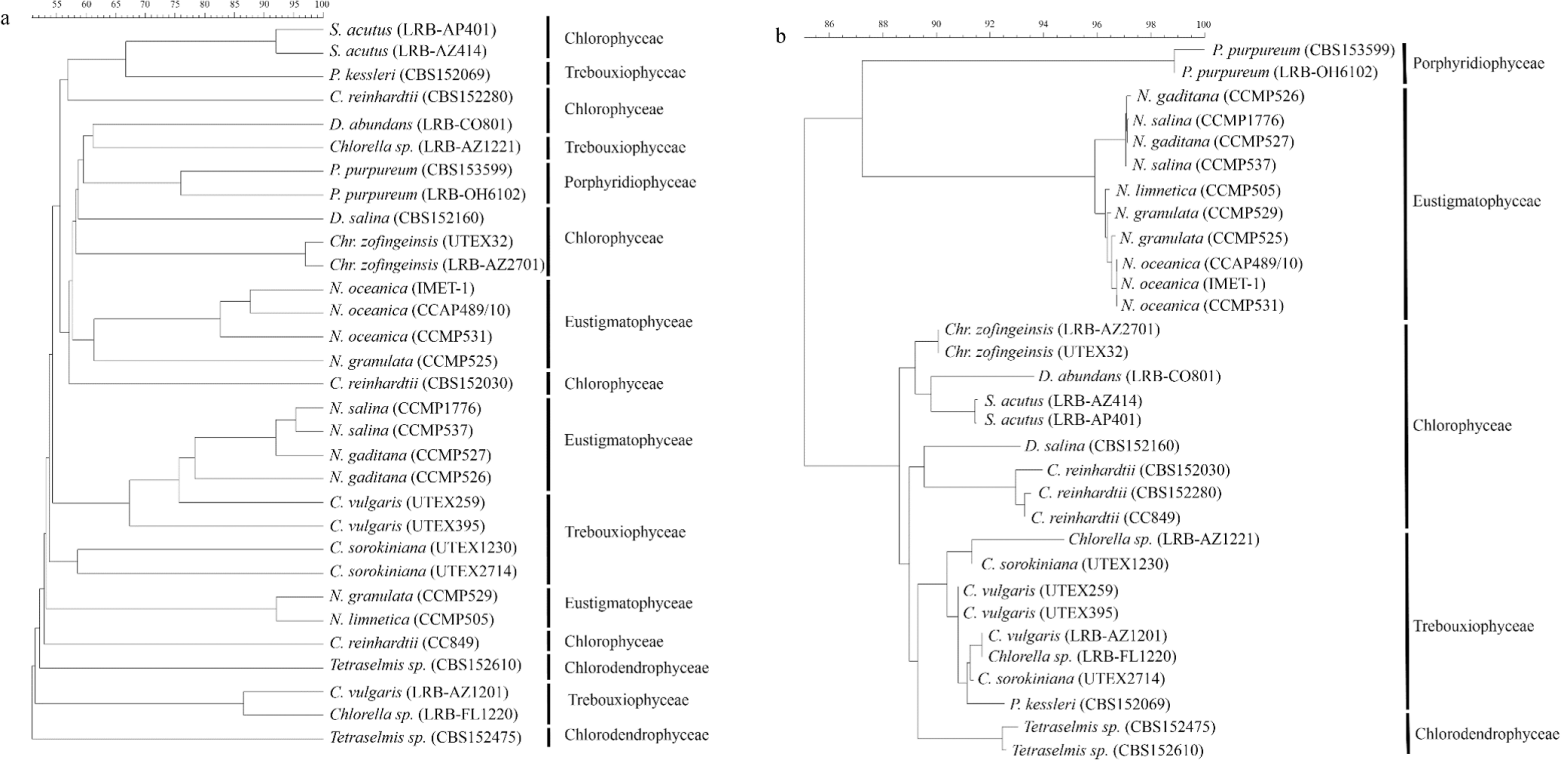
Similarity-based dendrograms representing spectra (a) and 18S rDNA sequences (b) of 31 microalgae. Class names are listed on the right to facilitate comparison between the two dendrograms. The 18S rDNA sequence-based dendrogram (b) shows grouping of the samples up to the class level. Spectra (a) were clustered using the UPGMA algorithm, while sequences (b) were clustered using the neighbor joining algorithm.

Differences observed in the spectra summarized above were reflected in the cluster analysis of spectra of all 31 microalgae examined here (Fig 3A). Spectra of microalgae clearly separated at the species level. Separation of microalgae at the strain-level was also observed (Fig 3A).

We also performed 18S rDNA sequence analysis and compared it to the MALDI-TOF MS data. The 18S rDNA-based dendrogram (Fig 3B) included five clades corresponding to five classes of microalgae represented in our collection. As expected, members of the same genus and species clustered together; however, at the strain level, the 18S rDNA sequence data did not afford clear separation of *Nannochloropsis salina* strains and *Scenedesmus acutus* strains. *N*. *salina* CCMP1776 and CCMP537 sequences had no differences in 18S nucleotide sequences; *S*. *acutus* LRB-AP 401 and LRP-AZ 414 differed by only 2 nucleotides. Additional DNA sequencing data of regions such as ITS-2 would be required to clearly differentiate these strains. In contrast, the MS-based dendrogram (Fig 3A) clearly separated nearly all strains examined including strains of *N*. *salina*, *S*. *acutus*, *C*. *vulgaris*, *C*. *reinhardtii*, *N*. *oceanica*, *N*. *gaditana*, and *P*. *purpureum*. The dendrogram based on the 18S rDNA data demonstrated a much higher degree of taxonomic organization (i.e., members of the same class clustered together) compared to the MS-based dendrogram as has been reported previously [7]. Similar to our results, Lee et al. [7] reported intermixing of taxonomically similar microalgae at the class-level in an MS-based dendrogram. Differences between MALDI- and 18S rDNA sequence-based dendrograms are reflective of the facts that: 1) 18S rDNA dendrograms are based only on gene sequence data, while MALDI dendrograms and spectra contain proteome-level, gene expression-based data and 2) different clustering algorithms are routinely employed with each type of data (i.e., gene sequence data are typically clustered using the neighbor-joining algorithm [5], while MALDI spectra are often clustered using the Unweighted Pair Group Method with Arithmetic Average (UPGMA) algorithm [9]).

### Mixture Analysis

**Fig 4:**
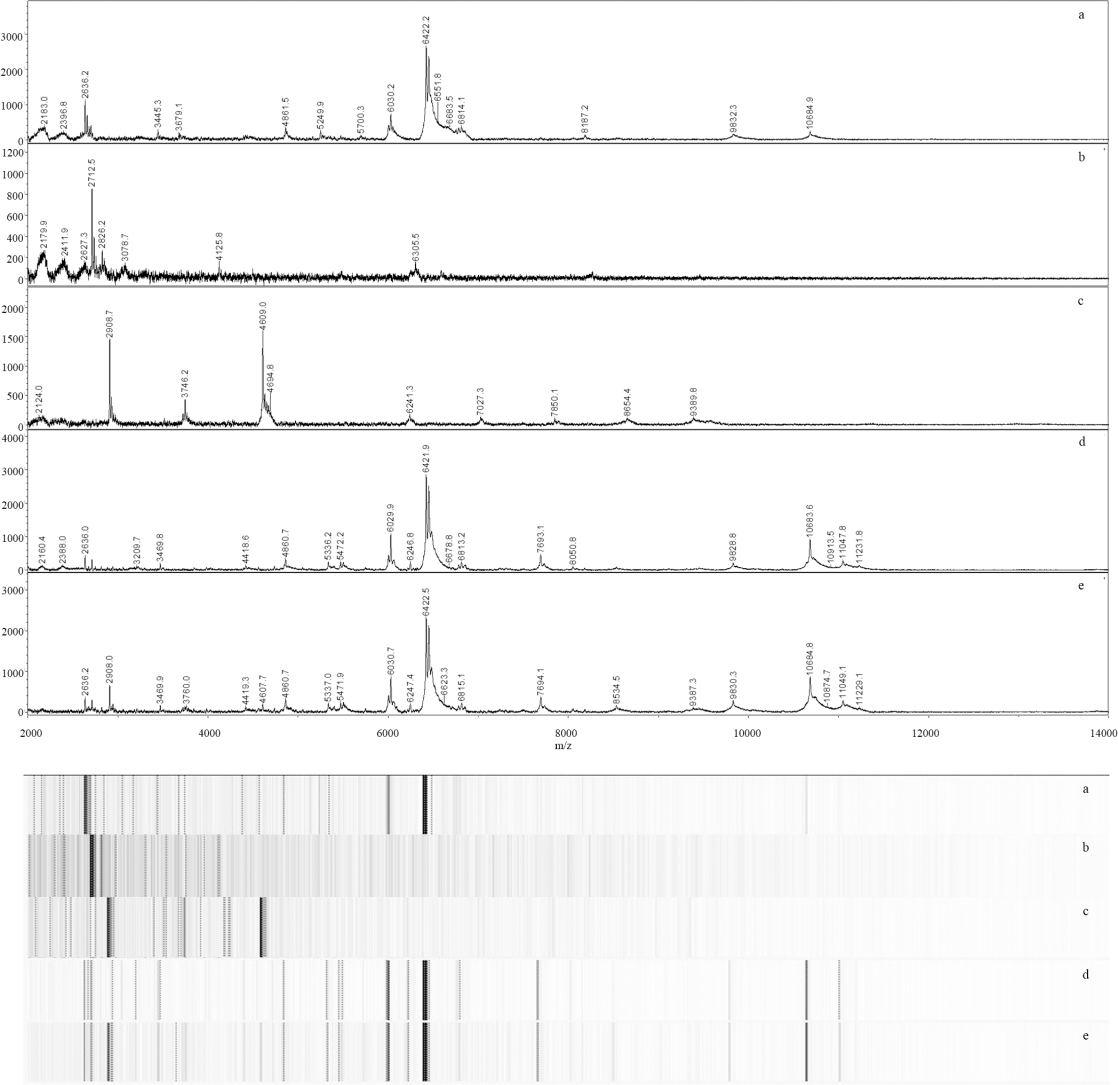
MALDI-TOF spectra and pseudo-gels of simple mixtures containing two or three individual microalgae. Representative spectra of samples of *Chlorella vulgaris* UTEX 395 (a), *Scenedesmus acutus* LRB-AP 401 (b), *Chlorella sorokiniana* UTEX 1230 (c), a mixture of *C*. *vulgaris* UTEX 395 and *S*. *acutus* LRB-AP 401 (d), and a mixture of all three microalgae (e).

Rapid detection of contaminating microalgae and deleterious community shifts are important during outdoor pond cultivation of microalgae biomass. For this reason, we attempted to use MALDI to characterize simple mixtures of microalgae. As has been reported frequently with MALDI analysis of bacterial mixtures, spectra of mixtures of microalgae contained many peaks originating from the individual microalgae composing the mixture [31–36]. The first mixture contained *C*. *vulgaris* UTEX 395 and *S*. *acutus* LRB-AP 401. Six prominent peaks from these two individual microalgae were observed in the spectrum of this mixture (Table 2; Fig 4). The second mixture contained *C*. *vulgaris* UTEX 395, *S*. *acutus* LRB-AP 401, and *C*. *sorokiniana* UTEX 1230. Spectra from this mixture contained eight prominent peaks found in the spectra of the constituent three individual microalgae.

**Table 2:** Peaks observed in spectra of individual microalgae and simple mixtures.

m/z	*C*. *vulgaris* UTEX 395(Sample 1)	*S*. *acutus* LRB-AP 401(Sample 13)	*C*. *sorokiniana*UTEX 1230(Sample 8)	Mixture 1 (Samples 1 & 13)	Mixture 2 (Samples 1, 8, & 13)
2636	M			M	M
2712		M		M	M
2735		P			
2908			M		M
4608			M		M
6030	M			M	M
6422	M			M	M
6451	M			M	M
6481				S	S
10684	M			M	M
11048				S	S
11230				S	S

M = peak from individual microalga that was observed in one or both mixtures

P = peak from individual microalga that was not observed in either mixture

S = mixture-specific peak

Not all peaks observed in spectra of individual microalgae were observed in the mixture spectra. Peaks present in the spectrum of *C*. *vulgaris* UTEX 395 dominated both mixture spectra (Table 2; Fig 4). As previously postulated [31], ion suppression may account for underrepresentation of individual microalgae in the spectra of mixtures. Ion suppression results when one analyte suppresses appearance of another in a mass spectrum due to: 1) the suppressing ion being present at a higher concentration than the suppressed ion and/or 2) the suppressed ion does not ionize as efficiently as the visible ion. We adjusted the OD_750_ of each microalgae sample to 0.3 before constructing the mixtures, but the *C*. *vulgaris* peaks remained the most prominent in the mixture spectra. It is possible that the *C*. *vulgaris* yielded more readily ionized proteins compared to *S*. *acutus*, but further work is warranted to further clarify mechanisms of peak suppression in microalgal mixtures.

Interestingly, spectra of both mixtures exhibited unique peaks at m/z 6,481; 11,048; and 11230. These three peaks appear to be mixture-specific as they do not appear in the spectra of the individual microalgae constituents. Similar results have been reported previously with bacterial mixtures [31], in which two mixture-specific peaks were observed in a mixed culture of *E*. *coli* and *S*. Typhimurium. The origin of these peaks and the mechanism of their formation is not clear, but may result from interactions between proteins (e.g., enzymes) associated with the individual cultures. Alternatively, interspecies interactions between the algae may have induced expression of proteins represented by these peaks. In either case, these mixture-specific peaks may provide information that is useful in the rapid characterization of algal mixtures and/or identification of contamination of algal cultures.

## Conclusions

Our results suggest that MALDI-TOF MS represents a rapid and effective alternative to conventional methods of characterizing microalgae. To our knowledge, this is the first report of the use of MALDI to characterize mixtures of microalgae (polycultures), which are gaining popularity within the microalgae production industry. The taxonomic resolution of this rapid approach appears superior to conventional gene-sequencing based methods, as has been reported recently with *Dunaliella* [20]. Mixture-specific peaks were observed and may serve as biomarkers of contamination that allow producers to rapidly detect contamination events. Accordingly, MALDI-TOF MS has potential as a more rapid and economical means of monitoring the health and productivity of microalgae culture systems. For this reason, our current efforts include development of sample preparation and data analysis workflows that facilitate rapid analysis of more complex microalgal mixtures, including those that result from contamination events and predator introduction.
